# Ultrasonic‐Enabled Nondestructive and Substrate‐Independent Liquid Metal Ink Sintering

**DOI:** 10.1002/advs.202301292

**Published:** 2023-06-14

**Authors:** Sanhu Liu, Zhiwu Xu, Guoqiang Li, Zhengwei Li, Zihan Ye, Zirong Xu, Wenjun Chen, Dongdong Jin, Xing Ma

**Affiliations:** ^1^ State Key Laboratory of Advanced Welding and Joining Harbin Institute of Technology Harbin 150001 China; ^2^ School of Materials Science and Engineering Harbin Institute of Technology Harbin 150001 China; ^3^ Sauvage Laboratory for Smart Materials School of Materials Science and Engineering Harbin Institute of Technology (Shenzhen) Shenzhen Guangdong 518055 China

**Keywords:** circuits fabrication, flexible electronics, liquid metal, non‐contact sintering, ultrasonic sintering

## Abstract

Printing or patterning particle‐based liquid metal (LM) ink is a good strategy to overcome poor wettability of LM for its circuits’ preparation in flexible and printed electronics. Subsequently, a crucial step is to recover conductivity of LM circuits consisting of insulating LM micro/nano‐particles. However, most widely used mechanical sintering methods based on hard contact such as pressing, may not be able to contact the LM patterns' whole surface conformally, leading to insufficient sintering in some areas. Hard contact may also break delicate shapes of the printed patterns. Hereby, an ultrasonic‐assisted sintering strategy that can not only preserve original morphology of the LM circuits but also sinter circuits on various substrates of complex surface topography is proposed. The influencing factors of the ultrasonic sintering are investigated empirically and interpreted with theoretical understanding by simulation. LM circuits encapsulated inside soft elastomer are successfully sintered, proving feasibility in constructing stretchable or flexible electronics. By using water as energy transmission medium, remote sintering without any direct contact with substrate is achieved, which greatly protect LM circuits from mechanical damage. In virtue of such remote and non‐contact manipulation manner, the ultrasonic sintering strategy would greatly advance the fabrication and application scenarios of LM electronics.

## Graphical abstract

An ultrasonic sintering strategy is developed for recovering conductivity of liquid metal (LM) ink circuits on various substrates, which can be readily used to fabricate LM based printed and flexible electronics, for those patterned on complex or curved surfaces.

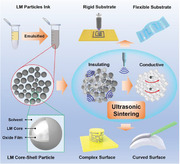

## 1 Introduction

Gallium based liquid metal (LM) has significantly advanced the development of soft and flexible electronics that are used in various emerging fields, such as electronic skins,^[^ ^1^ ^]^ implantable devices,^[^ ^2^ ^]^ triboelectric nanogenerators,^[^ ^3^ ^]^ soft robotics^[^ ^4^ ^]^ and flexible sensors.^[^ ^5^ ^]^ Numerous methods have been reported and used to pattern LM circuits, including injection,^[^ ^6^ ^]^ additive methods,^[^ ^3^, ^7^ ^]^ subtractive methods^[^ ^8^ ^]^ and lithography.^[^ ^9^ ^]^ However, the high surface tension of LM hinders its direct printing or coating on a variety of substrates. Therefore, LM ink composed of surfactant stabilized LM micro/nano‐particles has been developed,^[^ ^10^ ^]^ which can adhere on most substrates and realize personalized printing of LM based electronics. This strategy greatly improves the efficiency and adaptability of LM ink based electronic devices and has been applied in a wide range of fields such as motion monitoring,^[^ ^11^ ^]^ biosensing^[^ ^12^ ^]^ and electrical stimulating.^[^ ^13^ ^]^

The presence of oxide film on the external shell associated with surfactant molecules makes LM particles insulating. To obtain a conductive path, it is essential to break the oxide shell and make the fluidic core flow out and fuse together to electronically connect LM particles with each other. Mechanical sintering, including pressing and stretching the as‐prepared LM ink based circuits, is mostly applied to break the oxide shell and obtain conductive LM circuits.^[^ ^10b,d^ ^]^ However, several problems remain challenging for current mechanical sintering. First, the flowing LM may have contact with adjacent circuits or electrical components to cause a short circuit due to the low precision of mechanical manipulation process. Besides, the external force directly applied on the electronic devices may damage the encapsulation elastomer and even induce the leakage of LM. Furthermore, mechanical sintering is not applicable when patterning LM ink on rough and complex surfaces (e.g., curved, grooved, etc.), as conventional pressing tools (e.g., rollers) cannot reach all the surfaces of the LM ink patterned on various substrates like those with asperities. Thus, laser sintering^[^ ^14^ ^]^ and self‐sintering^[^ ^15^ ^]^ of LM ink have been proposed to solve the above‐mentioned problems. When LM particles are exposed to laser, thermal expansion of LM core induced by photothermal effect can break the oxide shell, and then conductive path will be constructed in laser treated area to achieve high‐resolution LM circuits. However, LM ink in complex surfaces including grooves, corners and pores is difficult to obtain enough energy due to reflection and scattering of light. Moreover, the heat from laser may ablate soft substrate and lead to deformation or damage of electronics. The self‐sintering method utilizes the expansion of polymers derived from swelling or capillary force to rupture the oxide film, which, however, requires a long‐time water evaporation process, and thus seriously deteriorates the fabrication efficiency. To address these problems, fast and facile manufacture to sinter LM ink patterns is highly demanded, especially for those patterns on substrates with complex surface structures.

**Scheme 1:**
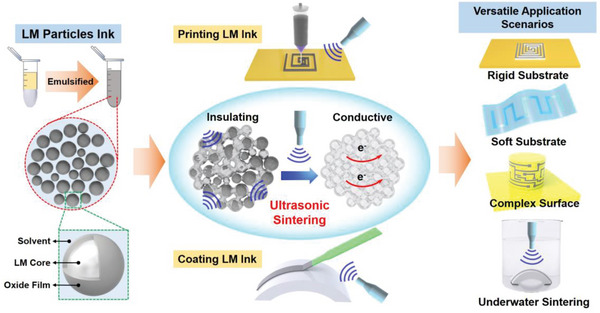
Schematic illustration showing preparation of LM particles ink, and ultrasonic sintering of LM ink circuits on various substrates for the fabrication of flexible &printed electronics in versatile applications scenarios.

Here, we present an ultrasonic sintering strategy for constructing conductive circuits on various substrates from pre‐patterned LM particles‐based ink, which can protect LM circuits and build conductive patterns on various substrates and surfaces (**Scheme** **1**). The ultrasonic horn and a resonating unit provide ultrasonic vibrations, friction and heat are generated by mechanical vibration from the horn so that two joints can be welded. With a short‐time ultrasonic treatment, conductive LM circuits can be obtained quickly on both rigid and soft substrates. The shape of the LM ink pattern can be well preserved compared with the conductive circuits achieved by mechanical sintering. By analyzing the effect of ultrasonic parameters and LM ink micromorphology, an ultrasonic enabled facile and efficient method has been developed to fabricate LM based electronics. Flexible pressure/strain sensors with long‐term stability performance are fabricated, which demonstrate high reliability of the LM circuits. Furthermore, we demonstrate ultrasonic sintering on rough or grooved surface where LM ink pattern cannot be mechanical sintered at all. Meanwhile, ultrasonic sintering underwater is also successfully realized by applying ultrasound in a remote manner, which demonstrates the wide adaptability potential of the ultrasonic sintering strategy, such as sintering on 3D topographical surface. The presented ultrasonic assisted sintering would greatly expand the application scenarios of the LM ink based flexible and printed electronics. Furthermore, it would also inspire future advancement on non‐contacting manipulation of the LM based materials or patterned structures.

## 2 Results and Discussions

### 2.1 LM Ink Preparation and Ultrasonic Sintering on Rigid Substrate

**Figure 1:**
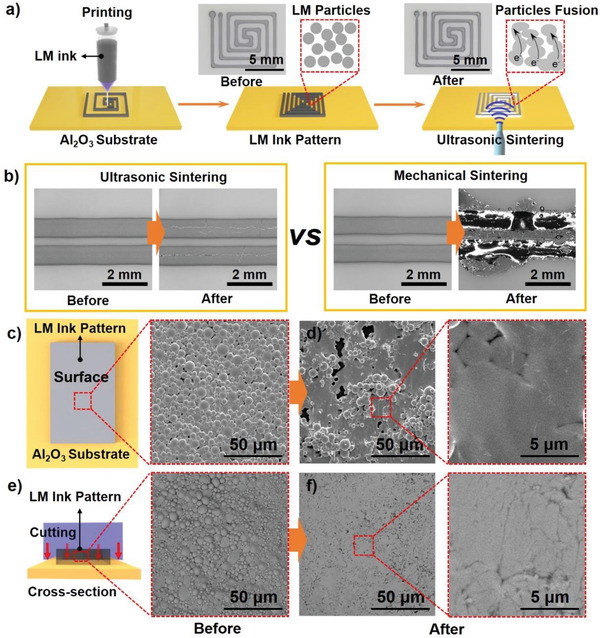
Ultrasonic sintering of LM ink pattern on rigid Al_2_O_3_ board. a) Scheme of LM ink patterning and ultrasonic sintering process of the printed LM ink. b) Comparation of the LM ink patterns between those treated by ultrasonic sintering and mechanical sintering. c) Schematic illustration of the SEM observation on the surface of LM ink pattern and SEM image of LM ink surface before ultrasonic sintering. d) SEM image of LM ink surface after ultrasonic sintering. e) Schematic illustration of the SEM observation on the cross section of the LM ink pattern and cross section SEM of LM ink before ultrasonic sintering. The LM ink circuit was cut after being frozen at −80 °C for 5 min. f) SEM image of LM ink cross section after ultrasonic sintering.

The LM particles‐based ink was synthesized by emulsion treatment of bulk LM in ethanol according to our previous study.^[^ ^10a^ ^]^ Polyvinylpyrrolidone (PVP) was added as the surfactant to stabilize the formed LM particles, denoted as LMP@PVP (Figure S1a, Supporting Information). The obtained LM ink was then transferred into a syringe for printing circuits by an electronic printer (Figure S1b, Supporting Information). With the addition of PVP, LM particles can maintain well dispersed state, as shown in Figure S2 (Supporting Information). Subsequently, we printed LM ink on the Al_2_O_3_ board. The ultrasonic sintering process is illustrated in **Figure** **1**a. The frequency of applied ultrasound was 30 kHz. The printed circuit consisted of plenty of LM particles that were wrapped with oxide film and PVP molecules. Therefore, the whole circuits were insulating. We then applied an ultrasonic horn on the bottom of the Al_2_O_3_ board. It could be found that metal luster appeared after applying ultrasound, indicating the rupture of oxide layer and formation of conductive paths (Figure 1b). With the assistance of ultrasound, LM particles were broken and connected with each other, which was denoted as “ultrasonic sintering”. Compared with mechanical sintering, ultrasonic sintering can maintain the shape of circuits and break the oxide film without touching the circuits. We also printed a LM ink circuit to demonstrate the “shape protection” by ultrasonic sintering as compared to the apparent destruction induced by mechanical sintering (Figure 1b). During mechanical sintering process, LM ink circuits were pressed by a piece of quartz glass to break the oxide film. When the oxide film was ruptured, LM flowed out and the original pattern was changed, along with apparent damage to the circuits. Conversely, the circuits shape remained almost intact after we applied ultrasound on the board. Therefore, conductive LM circuits with different width could be fabricated easily through printing of LM ink and ultrasonic sintering (Figure S3, Supporting Information). Besides, conductivity of LM ink with different content of PVP after mechanical sintering and ultrasonic sintering was compared. Increasing the polymers content of LM ink will decrease the conductivity of sintered LM ink. Although the polymers in LM ink can stabilize the LM particles and maintain the LM ink shape, they may also induce failure of mechanical sintering. As shown in Figure S4 (Supporting Information), we prepared 4 groups LM ink with different PVP content. M_LM_ and M_PVP_ represent the mass of LM and PVP, respectively. When the LM ink contains the lower content of polymeric surfactant, e.g. M_LM_ : M_PVP_ = 60 : 1, both mechanical sintering and ultrasonic sintering work well. With PVP increasing, for the group M_LM_ : M_PVP_ = 5 : 1, LM ink was insulating after mechanical sintering but became conductive after ultrasonic sintering. The result indicates that ultrasonic sintering is a more effective sintering method especially when the LM ink particles were wrapped with high content of surfactants. Considering the important role of these polymeric surfactants, e.g., PVP, for LM ink preparation and their colloidal stability, such an advantage of higher sintering efficacy will greatly extend the application of LM ink in many printing electronics.

Micromorphology of the LM ink pattern before and after ultrasonic sintering was also observed to certify the sintering process (Figure 1c–f). First, we investigated the top surface of the circuits (Figure 1c). The LM circuits for scanning electron microscope (SEM) observation were prepared after being frozen at −80 °C for 5 min, preventing the probable damage (e.g., random flow of LM change of LM circuits, etc.) in the circuits removing process. Before ultrasonic sintering, LM particles were in contact with each other, but separated by a thin shell of oxide film and the gaps between particles (Figure 1d). After ultrasonic sintering, most LM particles were broken, the liquidous LM inside flowed out and formed a conductive path. According to previous study,^[^ ^10a^ ^]^ some residual oxide and PVP molecules may wrap around these particles, which probably provide adhesion force to preserve the shape of the circuits. Then we cut the circuits and observed the cross section of the LM ink pattern. The sample was also frozen at −80 °C to maintain solid state before cutting. Separated LM particles could be found before sintering (Figure 1e), indicating the cutting process did not damage the original morphology of particles. SEM image of the cross‐section view of the LM ink after ultrasonic sintering was different from that of the surface (Figure 1d). Separated LM particles could hardly be found, while bulk LM with some wrinkles and small voids appeared instead. Besides, wrinkles in Figure 1f showed the collapse and connection of the LM particles, which was the clear evidence for the ultrasound induced strong vibration and fusion of these particles. We speculated that mechanical effect of ultrasonic vibration broke the oxide film, thus realizing sintering of LM ink on rigid substrate. Since the particle break and the connection of LM is the common mechanism of sintering of LM particle‐based ink. Ultrasonic sintering can also be applied to other LM ink without PVP. Vibration induced by ultrasonic waves is similar in LM particles, which can break the oxide film wrapped LM particles and construct conductive path. Meanwhile, due to the high surface tension and tendency to oxidation of EGaIn, bulk LM lines are formed by spheres surrounded by oxide that prevent them from forming good electrical contact. Ultrasonic sintering can also be used to break the oxide shell and make the LM flow in between to bridge electrically the whole line. These results confirmed the feasibility of ultrasonic sintering of LM ink pattern, which encouraged us to further study the mechanism and application of the ultrasonic sintering strategy for electronic devices fabrication.

### 2.2 Ultrasonic Sintering of LM Ink Circuits on Flexible Substrate

Ultrasonic treatment of LM particles has very recently been reported,^[^ ^16^ ^]^ which demonstrates the promising usefulness of ultrasonic technique in fabrication of LM based electronic devices. However, the conductive paths were achieved by sonication treatment of LM particles in polymeric elastomer instead of directly sintering of LM ink based conductive circuits patterned on on‐demand substrates. Details of the difference are listed in Table S1 (Supporting Information). The application of LM usually involves flexible electronics, which demands soft substrates and/or encapsulating stretchable materials. The success of ultrasonic sintering of LM ink circuit on rigid substrate encouraged us to expand this technique to flexible substrate. Ultrasonic soldering has been widely used in joining materials such as aluminum,^[^ ^17^ ^]^ magnesium^[^ ^18^ ^]^ and ceramics.^[^ ^19^ ^]^ The propagation of ultrasonic waves in materials could directly apply mechanical force to break the oxide film. Ultrasonic soldering, both in plastics and metals, permits the very delicate joining of components in virtue of precise control on the energy input and local pressure. However, attenuation of ultrasound is a nonnegligible problem in common soft polymers, such as the most widely used polydimethylsiloxane (PDMS) in flexible electronics. Based on our preliminary hypothesis about vibration mechanism of the ultrasonic sintering, transmitting enough mechanical vibration force to the LM ink is crucial for the success of the ultrasonic sintering.

**Figure 2:**
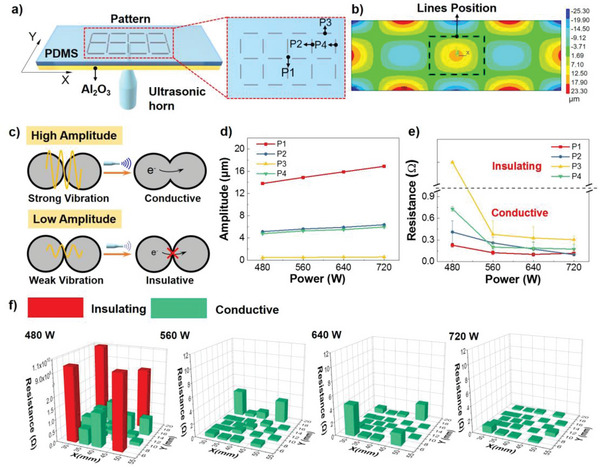
Investigation of ultrasonic sintering of LM ink lines on flexible substrate. a) Scheme of the experimental setup showing ultrasonic sintering of LM ink patterns printed on a PDMS layer. b) Ansys simulation of vibration amplitude distribution on the substrate. c) Schematic illustration of the ultrasonic sintering of LM particles under low and high amplitude of ultrasonic vibration. d) Amplitude on P1‐P4 from Ansys simulation results. e) Resistance of LM ink lines on P1‐P4 after treated with different ultrasonic power (error bars: SD, *n* = 3). f) Resistance of different LM ink lines showed in a) after treated with different ultrasonic power. The ultrasonic horn is at the center of Al_2_O_3_ board. Green columns represent conductive lines and red columns represent insulating lines.

Thus, we designed a 3‐layer structure consisting of an Al_2_O_3_ board, thin PDMS film and LM ink line on top. Thickness of the PDMS layer was only ≈60 µm to ensure sufficient penetration of ultrasonic energy for sintering the LM ink line. To study the performance of ultrasonic sintering, 22 lines were printed on the substrates (**Figure** **2**a) and the electrical resistance of each line was measured. The ultrasonic horn was placed on the bottom of Al_2_O_3_ board (below P1). The frequency of applied ultrasound was 30 kHz. Ansys simulation was used to analyze the vibration on the Al_2_O_3_ board. The thin PDMS film was spin‐coated on the Al_2_O_3_ board and ultrasonic horn was applied on the bottom of Al_2_O_3_ board. Details of the LM lines position was plotted in Figure S5 (Supporting Information). The simulated amplitude distribution of the mechanical vibration induced by the ultrasonic treatment was plotted in Figure 2b and Figure S6 (Supporting Information). There were several maximum points on the board, including the center and the edge. To explore the amplitude variation versus applied power of the ultrasound, we chose four feature points: P1, P2, P3, P4, and calculated their amplitudes under different ultrasonic powers. As shown in Figure 2d, amplitude of P1 presented an obvious increase with increasing ultrasonic power. However, amplitude in P3 remained at ≈0.5 µm while the applied power increased from 480 to 720 W, indicating that vibration around P3 might be too small to break the oxide film. We speculated that the vibration on the board broke the film around LM particles, thus forming a conductive path (Figure 2c). Once the ultrasound was applied, high amplitude vibration with high amplitude can break the oxide film. While in low amplitude area, oxide film could not be broken, and LM ink lines were still insulating. P3 was insulated under 480 W (Figure 2e).

As shown in Figure 2f, 4 out of 22 lines were insulating under 480 W and the distance from their location to the ultrasonic probe was almost same to that of P3 point. The rest 18 lines, which located in the center area of the ultrasonic probe were all conductive from 480 to 720 W. These results were consistent with the simulation results (Figure 2b). As the results showed in Figure 2d, the vibration amplitude of P3 stayed unchanged with increasing input power. However, with the increase of ultrasonic power, all the 22 lines became conductive. Thus, considering the properties of ultrasound vibration, we suspected that ultrasonic sintering was influenced by not only the vibration of single point but also the vibration status around the point. Taking P3 as an example, P3 was a feature point just on the center of a line. The line was located on the green region where the vibration amplitude ranged from 1.69 to −3.71 µm (Figure 2b). It should be noticed that the increase of ultrasonic power would expand this range and relative high amplitude would appear on the edge of the line. In such an ultrasonic field, LM would move from high amplitude area to low amplitude area, squeezing other particles and breaking the oxide film and thus making the whole line conductive.

The position of ultrasonic horn would influence the vibration distribution.^[^ ^20^ ^]^ We prepared the same Al_2_O_3_ boards as Figure 2a and printed 9 LM ink lines. Then, we evaluated the sintering result while applying ultrasonic horn at 4 different positions. The position details of lines and ultrasonic horn's location were shown in Figure S7' (Supporting Information). We used “X” to represent the distance between ultrasonic horn and the board's long side edge. In our experiment, we set 4 different distances (X1 = 3 mm, X2 = 6 mm, X3 = 9 mm, X4 = 12.5 mm) as shown in Figures S9–S12 (Supporting Information). LM ink lines were numbered “1‐9” and conductivity of each line was measured after ultrasonic sintering (Figures S8–S12, Supporting Information). We prepared 3 samples for each condition and plot the result in Figures S9–S12 (Supporting Information). Although the conductivity change was not very regular, we could still find some trends. First, when the power was 480 W No. 4 lines became conductive when the ultrasonic horn was placed right in the center (X = 12.5 mm). Second, with the increase of ultrasonic power, more lines would be sintered, which indicated an increase of vibration. Third, when we applied the ultrasonic horn on the edge of Al_2_O_3_ boards (X = 3 mm, 6 mm), most lines at edge (No. 1 – 3, No. 7 – 9) were sintered. When the ultrasonic horn was close to center (X = 9 mm), we could find that lines at edge were not sintered, which indicated the reduction of vibration. In addition, higher power input (720 W) would induce higher vibration on the whole board, which could effectively sinter LM ink both at edge and in the center. However, it should be noticed that not all sintering results followed the same rule, which might be attributed to the fluctuation of frequency and attenuation of ultrasound. More detailed discussions can be seen in Note S1 (Supporting Information). Thus, further investigation is needed to analyze interface structure and ultrasonic waves resonance of different materials to clarify more in‐depth and fundamental mechanism of ultrasonic sintering of LM ink.

### 2.3 Impact of Ultrasonic Time, PDMS Thickness and LM Particle Size on the Sintering Efficacy

In addition to ultrasonic energy and ultrasonic horn position, other influencing factors also affect the ultrasonic sintering efficacy, including ultrasonic time, particle size and PDMS thickness. We further investigated the influence of these parameters and optimized them. The frequency of applied ultrasound was 30 kHz in these experiments. It is worth mentioning that long‐time ultrasonic time would cause circuits damage and LM leakage, so ultrasonic time should be properly controlled. To in situ observe the microstructure evolution of the LM ink pattern under ultrasonic sintering, a platform equipped with charge‐coupled device (CCD) and microscope was constructed (Figure S13, Supporting Information). We used a transparent glass as substrate, in which way we could directly visualize the microstructure of the coated LM ink pattern on the glass substrate from both front and back sides. Elastic modulus and Poisson's ratio of glass were different from that of Al_2_O_3_ board, which would change the vibration distribution and sintering results. However, the vibration amplitude at the substrate center was high enough to sinter the LM ink when using 720 W ultrasonic power. Observing the sintering process on glass could provide us detailed information about micromorphology's evolution to optimize ultrasonic time (Figure S14, Supporting Information). Metal luster appeared on both sides of sintered LM ink (Figure S15, Supporting Information), which further confirmed that LM inside flowed out after ultrasonic treatment. While applying ultrasound, the CCD would capture the movement of LM ink. As shown in Video S1 (Supporting Information), cracks gradually appeared on the surface and LM flowed out. The LM began to connect with each other with ultrasonic time increasing. After 2 s ultrasonic sintering, more cracks could be observed. It should be noticed that cracks appearance implied the damage to the shape of circuits. Besides, overflow LM would increase the risk of short circuit. Thus, the ultrasonic time was optimized to be within 2 s.

**Figure 3:**
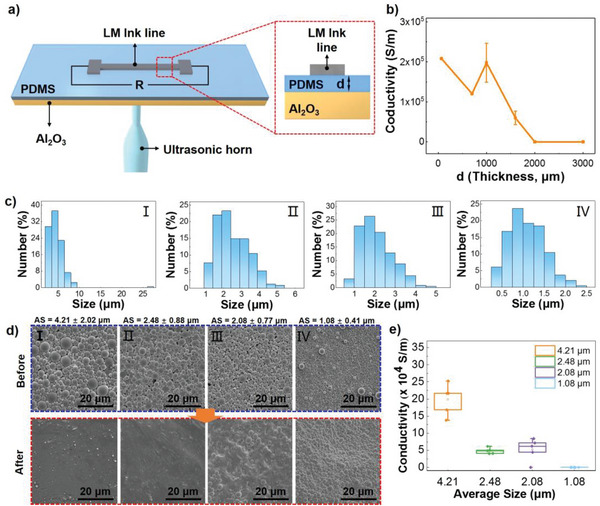
Impact of sintering parameters on the ultrasonic sintering efficacy of LM ink circuits on flexible substrate. a) Schematic illustration of ultrasonic sintering of LM ink on PDMS substrate. b) Conductivity of LM ink line on PDMS substrate with different thickness after ultrasonic sintering (error bars: SD, *n* = 5). c) Size distribution of the LM ink particles. The ink was prepared by probe sonication for (I) 30 s, (II) 1 min, (III) 5 min, (IV) 10 min. d) SEM image of LM ink particles before and after ultrasonic sintering (720 W, 2 s). The AS of LM ink group I to IV is 4.21 ± 2.02, 2.48 ± 0.88, 2.08 ± 0.77 and 1.08 ± 0.41 µm (*n* = 200), respectively. e) Conductivity of the four group of LM ink lines after ultrasonic sintering (error bars: SD, *n* = 5).

The attenuation of ultrasound is related to the thickness of the soft PDMS layer, which directly influences the sintering efficacy of LM ink circuits. Generally, a thinner PDMS film will be better due to less attenuation. However, thin film is broken easily while being peeled or stretched. We need to find a proper thickness for the further fabrication of LM based flexible electronics. **Figure** **3**a illustrated the sample structure of the LM ink line for the ultrasonic sintering study. LM inks were printed on PDMS layers with different thickness that was precisely controlled by spin coating parameters. The ultrasonic horn was placed at the center of whole sample. Conductivity of the circuits were measured after ultrasonic sintering (720 W, 2 s), as shown in Figure 3b. We found that the sintered LM lines were still conductive until the PDMS thickness increased to 2 mm. The lines resistance after sintering increased dramatically when the PDMS thickness was increased to 1 mm, indicating apparent attenuation of ultrasonic energy in the soft PDMS layer. We observed larger deviation of resistance for larger thickness, which was probably caused by fluctuation of the ultrasonic waves. Considering the mechanical strength of the PDMS film and conductivity of circuits, the PDMS thickness was determined to lie in the range of 50–100 µm for the following flexible electronics fabrication.

Furthermore, the impact of the size distribution of LM ink was investigated to explore the possible LM particle size limit of the ultrasonic sintering method. Four groups of LM ink with different particle sizes were prepared and printed on PDMS substrate. The average particle size (AS) of the LM ink was calculated from SEM images in Figure 3d, which was 4.21 ± 2.02, 2.48 ± 0.88, 2.08 ± 0.77 and 1.08 ± 0.41 µm (average ± standard deviation (SD), *n* = 200) for group I to IV, respectively. Ultrasonic parameters and LM ink lines position were the same with those used in Figure 3b. Figure 3e illustrated the SEM images of LM ink before and after ultrasonic sintering. LM ink in group I to III could be sintered. When the AS decreased to 1.08 µm (group IV), LM particles could not be broken, indicating a size limitation effect for the ultrasonic sintering method. Moreover, the conductivity of LM ink line in each group was shown in Figure 3e, confirming that the circuits were insulating when average particle size was 1.08 µm. When the particle size was small enough (≈1 µm), ultrasonic wave was unable to cause resonance of LM particles so that LM ink could not be sintered. Therefore, LM ink with AS larger than 1 µm was needed for ultrasonic sintering in current setup. However, large particle size was unsuitable for precise printing and might bring damage to the circuits. In LM ink with larger AS (4.21 µm), we could find some particles over 20 µm in the ink. These particles were easily broken, and LM inside could flow out to the circuit edge, connecting with nearby circuits. Such results would bring damage to the shape of circuits and lead to short circuit, which was undesired in ultrasonic sintering. Although larger particles were more easy to be sintered with high conductivity after sintering, lager particles might also clog the needle, bringing additional troubles to the printing process. Therefore, LM inks with ≈2 µm particle size, *e. g*. group II, III in Figure 3c, were chosen as the optimal candidate for the ultrasonic sintering assisted fabrication of LM ink based conductive circuits.

### 2.4 Ultrasonic Sintering Assisted Fabrication of LM Flexible Electronics

**Figure 4:**
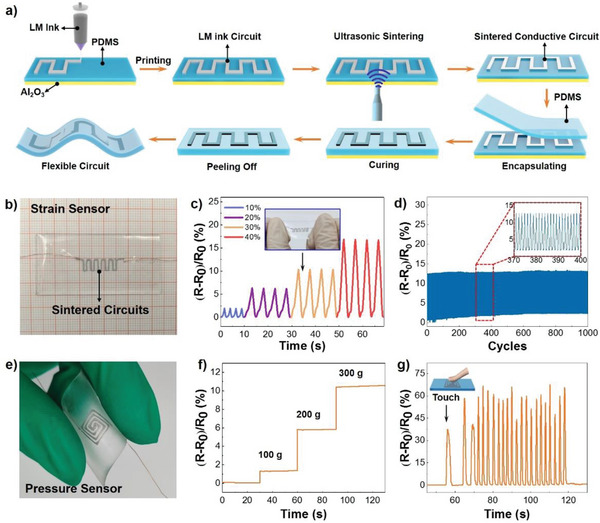
Ultrasonic sintering of LM ink circuits for flexible electronics fabrication. a) Scheme of ultrasonic sintering assisted fabrication of LM circuits based flexible electronics. b) Photograph of an as‐prepared LM flexible strain sensor. c) Relative resistance of the strain sensor in response to different tensile strains. d) Relative resistance response of the strain sensor under tensile strain during 1000 cycles, where the maximum tensile strain is 30%. e) Photograph of an as‐prepared LM pressure sensor. f) Relative resistance response of the pressure sensor under different pressures, where the sensor is loaded with different weights. g) Relative resistance response of the pressure sensor under random finger pressing.

Based on the optimized parameters, we developed an ultrasound sintering assisted fabrication method to prepare LM based flexible electronics as illustrated in **Figure** **4**a. The LM ink was directly printed on a PDMS layer that was spin‐coated on an Al_2_O_3_ board. Then, we placed an ultrasonic horn on the bottom of the Al_2_O_3_ board to apply ultrasound treatment and make the whole circuit conductive. The frequency of applied ultrasound was 30 kHz. The sintered LM circuit was further encapsulated in another new layer of PDMS before we peeled off the whole LM based flexible electronic device. Intrinsic stretchability of LM and encapsulated elastomer enabled these LM based flexible electronics with different functions.

Hereby, we demonstrated LM circuits‐based strain sensors and pressure sensors prepared by the ultrasonic sintering assisted fabrication strategy (Figure 4b–g). Photograph of the strain sensor device was shown in Figure 4b. Figure 4c depicted the relative resistance change ((R‐R_0_)/R_0_) under different stain, which kept increasing as the strain increased. Meanwhile, the strain sensor could hold an outstanding cycle stability. The relative resistance remained unchanged after 1000 cycles of 30% strain. These experimental data showed that strain sensor fabricated by ultrasonic sintering had excellent repeatability and consistency, which was essential for practical applications. Moreover, pressure change could be detected by monitoring the resistance change of the LM based circuits. Designed pattern of LM ink circuit was ultrasonic sintered and encapsulated inside PDMS, as shown in Figure 4e. While being pressed, the cross section of the circuit would be squeezed, resulting in resistance variation. Figure 4f showed relative resistance change of the LM circuit‐based pressure sensor while being pressed under different weights. In addition, the sensor exhibited only small fluctuations for the resistance change amplitude during 1000 cycles of repeated pressing and releasing at a weight of 100 g (Figure S16, Supporting Information). We could observe a dramatic rise of resistance after loading different weights. Such pressure sensor could be used to monitor the finger touch by analyzing the relative resistance response, as shown in Figure 4g. The fabrication of conductive patterns on soft substrates proves that ultrasonic sintering has the potential to construct flexible electronics.

### 2.5 Adaptability and Versatility of the Ultrasonic Sintering of LM Ink Circuits

To further demonstrate the adaptability and versatility of the strategy, ultrasonic sintering of LM ink circuits underwater and on rough/curved surface were carried out, all of which could hardly be realized by conventional mechanical sintering.

**Figure 5:**
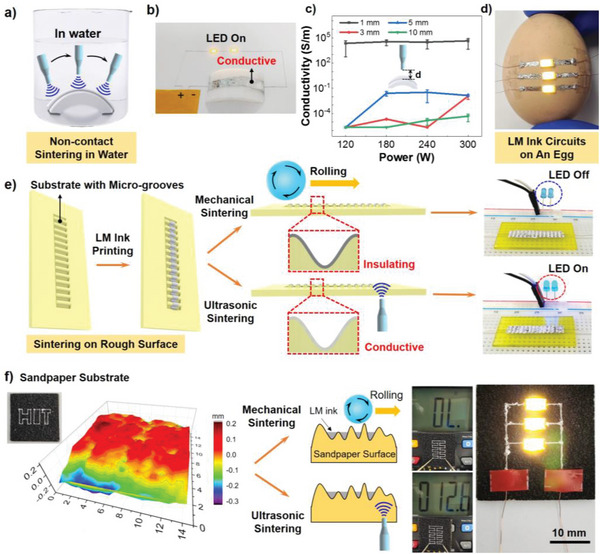
Ultrasonic sintering of LM ink circuits underwater and on rough/curved surfaces. a) Scheme of experimental setup showing ultrasonic sintering process underwater and conductivity of LM ink circuit. b) Photograph of the LED circuit used to demonstrate the conductivity of the sintered LM ink circuit. LM ink circuit was printed on a dome‐shaped sample holder and connected with LED and DC power, here the LED is on proving that the LM ink was sintered. c) Conductivity of the sintered circuit under water with different ultrasonic power and sintering distance “d” (error bars: SD, *n* = 5). d) LM ink circuits coated on an egg. The ink was ultrasonic sintered underwater, and LED was on. e) Schematic illustration of ultrasonic sintering on rough surface with grooves. The LED was lit on after ultrasonic sintering while mechanical sintering could not work. f) Surface profile of sandpaper and conductive LM ink lines on sandpaper fabricated by ultrasonic sintering. The average roughness (Sa) of sandpaper was 71.7 µm.

As shown in **Figure** **5**a, we first prepared a dome‐shaped sample holder by 3D printing and coated LM ink line on the curved top side surface. It was difficult for ultrasonic horn to be placed in fully contact with its upper curved surface, leading to the failure of ultrasonic sintering. Therefore, we proposed to introduce a media that could transfer the ultrasound energy to the LM circuit and break the LM particles for sintering. The good ultrasonic transmission property of water made it an idea candidate. We encapsulated the printed LM ink line with scotch tape and immersed the whole holder inside the water. Ultrasonic horn was placed above the LM ink with certain distance and moved along the circuit to sinter the whole circuit (Figure S17, Supporting Information). The frequency of applied ultrasound was 20 kHz. After applying the ultrasound treatment, we confirmed the conductivity of sintered ink through a light‐emitting diode (LED) circuit, where a DC power source, two LED lights and LM circuit were connected. LED was lightened up while being connected with the sintered LM circuit (Figure 5b), indicating that the LM circuit was conductive and proving the effectiveness of the ultrasonic sintering process under water. The phenomenon of acoustic cavitation would happen when ultrasound was applied directly inside water.^[^ ^21^ ^]^ Tiny bubbles generated by ultrasound in the compression phase of the ultrasonic wave would violently collapse, which could generate powerful mechanical shock wave to effectively break the oxide film on LM particles and thus realize sintering of LM ink circuit in water by remote ultrasound treatment.

Meanwhile, we investigated the influence of the distance between ultrasonic horn and LM ink circuit (denoted as sintering distance “d” shown in Figure 5c) on the sintering efficacy. Ultrasonic energy attenuates with the distance “d” increasing inside water. We measured circuits resistance after ultrasonic sintering underwater to explore threshold distance for effective sintering. As shown in Figure 5c, when the distance was 1 mm, the circuit was conductive with conductivity higher than 10^4^ S m^−1^. The increase of ultrasonic power did not reduce the resistance distinctly. When the distance increased to 3 mm, the conductivity was too low, and the circuit was insulating. We still found that the conductivity increased under an ultrasound power of 300 W and a distance of 3 mm, indicating a certain extent of sintering effect.

Moreover, when the distance increased to 5 mm, even 300 W ultrasonic power was not sufficient to break the LM particles. This phenomenon was attributed to the sound absorption by water. During the propagation of a plane sound wave through a medium, the intensity of the wave decreased as the distance from the ultrasonic source increased.^[^ ^22^ ^]^ The relationship between the intensity(*I*) and distance from the source (*d*) is given by:

(1) $$I\;=I_{0}\;exp\left(-2\alpha d\right)$$

where *α* is the absorption coefficient. The attenuation might arise because of reflection, refraction, diffraction or scattering of the wave. Moreover, some of the mechanical energy of the wave would be converted into heat, which might also contribute to the energy attenuation. It should be noticed that the distance between the ultrasonic horn and LM circuit was the key point for ultrasonic sintering underwater. In our experiment, the optimal distance was found to be ≈1 mm, for which ultrasonic energy could be effectively transported to the LM ink circuit for effective sintering. In addition, due to the ultrasonic energy absorption capacity of water, ultrasonic sintering underwater could protect fragile materials, which was verified using the LM ink circuits on an egg surface here. After ultrasonic sintering underwater, the LM ink circuits became conductive and the eggshell was not broken (Figure 5d), indicating that fragile substrates can be protected by ultrasonic sintering underwater. Nevertheless, we achieved sintering of LM ink circuit in a remote manner by using water as the ultrasonic energy transmission medium, which was very useful for sintering LM circuits patterned on curved surfaces.

Next, we demonstrated ultrasonic sintering on a wave‐shaped surface. The frequency of applied ultrasound was 30 kHz. Mechanical sintering usually demands direct and full contact with LM ink circuit to ensure exerting of external force on the whole circuit. Therefore, it is difficult for mechanical sintering to function on rough surfaces containing grooves or pores. Ultrasound waves can transmit in the substrate and break LM particles in the whole LM circuit, which makes it an effective method to solve this problem. We fabricated a board with grooves by 3D printing to prove its feasibility. As shown in Figure 5e, LM ink was coated on the whole surface and formed a circuit, which was insulating without sintering. LED was used to examine the conductivity of the whole circuit. While mechanically pressing the ink by finger touching, only the LM ink on the top of the grooves could be sintered, showing a little metallic luster. Without conductive path on the bottom of the grooves, the LED was maintained at off state. When applying ultrasound on the bottom of the board, LM ink both on the top and bottom of the grooves was sintered. The LED was on after the ultrasonic sintering, confirming the effective sintering of the LM ink circuit on grooved surface.

Directly patterning LM on a rough surface is difficult due to the limited contact between LM and substrate. Fluidic LM with large surface tension is unable to enter these surface asperities.^[^ ^23^ ^]^ Patterning LM particles ink on such rough surface can solve this problem because LM ink particles are able to adhere to different structures. However, effective sintering of LM ink on rough surface remains challenges and cannot be achieved by current mechanical sintering. LM ink in grooves cannot be touched and the whole LM ink line will remain insulating. We further used sandpaper to demonstrate ultrasonic sintering of LM ink on very rough surfaces. As shown in Figure 5f, LM ink line was printed on the sandpaper that was adhered to an acrylic board. Details of ultrasonic sintering on sandpaper were described in Experimental Section. LM ink on such rough surface cannot be mechanically sintered because LM ink in the grooves was difficult to be touched. When we applied ultrasound on the acrylic board, the LM ink became conductive. The surface profile of sandpaper surface was measured, and the result illustrated that LM ink conductive pattern can be fabricated by ultrasonic sintering can be applied on rough surface with maximum height of ≈200 µm.

**Figure 6:**
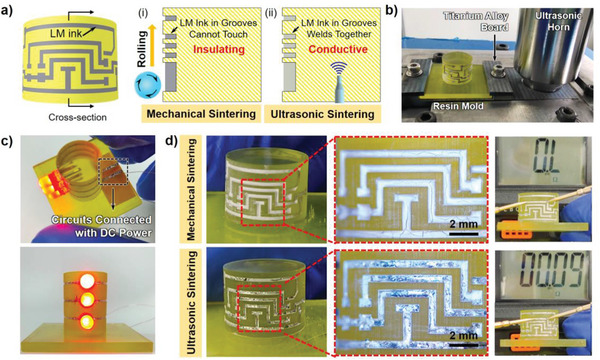
Ultrasonic sintering of LM ink patterns on 3D printed structure. a) Cross‐section view of resin mold and LM ink for mechanical sintering (i) and ultrasonic sintering (ii) in the grooves. b) The experimental setup for ultrasound assisted sintering of grooved LM circuits, which consisted of a resin mold fixed on titanium alloy board and an ultrasonic horn. c) Top view and front view of the LM circuits containing LEDs on a curved surface sintered by ultrasound. d) Complex LM ink circuits on the curved surface before and after ultrasonic sintering.

LM ink in square‐shaped groove could also be sintered, as shown in Figure S18 (Supporting Information). Besides, metallic luster on groove bottom proved that LM particles were broken (Figure S19, Supporting Information). Furthermore, conductive LM circuits with complex shapes could be constructed with the assistance of ultrasonic sintering, as shown in **Figure** **6**. The frequency of applied ultrasound was 20 kHz in these experiments. The yellow resin mold was fabricated by 3D printing. Grooves on the mold surface have advantages in protecting circuits and maintaining circuit precision. Obviously, mechanical sintering could not sinter LM ink in the grooves because external force was difficult to be applied on the bottom of grooves. While the mold was under ultrasonic vibration, LM ink circuits would become conductive since ultrasound wave could transmit in the material of the mold. To protect the mold, a titanium alloy board was used to fix the mold and assist the ultrasound transmission, as shown in Figure 5b. These results indicated that ultrasonic sintering was not only suitable for 2D circuits fabrication, but also a good candidate for LM based printed electronics on complex surfaces or even with 3D structure.

## 3 Conclusion

In summary, we report an ultrasound assisted sintering strategy for fabricating LM‐based flexible and printed electronics. Ultrasonic vibration can be applied on rigid substrate to break LM particles in the printed LM ink circuits and obtain conductive paths. By combing rigid Al_2_O_3_ board and flexible PDMS, we develop an ultrasonic sintering assisted method to fabricate LM based flexible electronics as demonstrated by strain and pressure sensors in current work. The shape of the LM ink circuit can be well protected through controlling the ultrasonic treatment conditions. By numerical simulation on the vibration amplitude distribution, we reveal that the sintering effect should be attributed to the ultrasound induced mechanical vibration that causes breaking and fusion of LM particles in the ink, although precise distribution of ultrasonic vibration on various substrates still needs further in‐depth investigation. Thanks to propagation of ultrasonic waves and cavitation effect in the liquid media, LM ink sintering underwater is realized, which provides a LM sintering technique in a remote manner. We further demonstrate the sintering of LM circuits on rough surfaces with grooves or curved surfaces, which is hard to achieve by normal mechanical pressing or stretching. This work enriches the fabrication techniques for LM based flexible and/or printed electronics, which will significantly facilitate the realization of LM‐based electronic applications in versatile practical scenarios.

## 4 Experimental Section

### Materials and Chemicals

Liquid metal (gallium 75 wt.% and indiun 25 wt.%) was purchased from Jilin Northeast Nonferrous Metals Company (China), PVP (> 98%) and Ethanol (EtOH, > 99%) were supplied by Aladin. Al_2_O_3_ board was purchased from Xien company (China). The PDMS (Sylgard 184, Dow Corning) prepolymer was prepared at a 10:1 oligomer/curing agent ratio.

### LM Ink Preparation

1 g PVP was added into 20 mL EtOH to prepare PVP solution. After that, 3 g eutectic Ga–indium (EGaIn) and 1 mL PVP solution were added in a centrifuge tube (5 mL). Subsequently, an ultrasonic Homogenizer (JY92‐IIDN) was used for sonication. The frequency of ultrasonic Homogenizer was 20 kHz. The power output of the ultrasonic system was first adjusted to 40% (240 W) and the sonication proceeded for 1 min. The temperature of the sample during sonication was controlled by cold‐water bath at ≈20 °C.

### Printing of LM Ink

LM ink printing was carried out using a microelectronic printer (Shanghai Mifang Technology Co., LTD.). LM inks were added in the syringes and digital pneumatic regulator was used for extrusion. The nozzle diameter was 250 µm and the pressure for printing was 150 kPa. Details of the printer are shown in Figure S1 (Supporting Information).

### Ultrasonic System

The ultrasonication system (HKD‐3030‐Q) was operated at a frequency of 30 kHz. The vibration direction was perpendicular to the sample surface.

### In Situ Observation of LM Ink

LM ink was directly coated on a glass (75 × 25 × 1 mm). A platform made of aluminum alloy was used to fix the glass (Figure S13, Supporting Information). There was a hole that allowed light transmission in the middle of the platform. An optical microscopy (NingBo Sunny Instruments Co., LTD.) with CCD was used to capture the images and videos. An ultrasonic horn was placed on the bottom of glass. When applying ultrasonic vibration, videos of LM ink motion could be recorded by CCD.

### LM Ink Morphology Characterization

LM ink was printed on the PDMS layer. Ultrasonic vibration was applied on the bottom of the Al_2_O_3_ board. After that, the sample was frozen at −80 °C for 10 min in a refrigerator (Thermo) to solidify the LM. Its can directly peel off the frozen ink by a tweezer from the substrate. To observe the cross section of LM ink, a blade was used to cut the LM ink. The cutting process was carried out within 30 s to prevent the melting. Images of surface morphology of samples were collected by scanning electron microscopy (Phenom Scientific) at 10 kV.

### Flexible Electronics Fabrication

Water‐soluble glue purchased from HORI company was coated on Al_2_O_3_ board. After drying in the air, PDMS was spin‐coated (750 r min^−1^, 60 s) on the glue layer. Subsequently, the sample was cured at 60 °C for 4 h. The LM ink was printed on the PDMS. Ultrasonic vibration (480 W, 2 s) was applied on the bottom of Al_2_O_3_ board to sinter the LM ink. PDMS were then poured onto LM ink circuit and cured at 60 °C for 4 h. The encapsulated sample was then immersed in water for 4 h to remove the glue layer. After that, sintered flexible electronics could be peeled off from Al_2_O_3_ board without damaging the circuit and PDMS layer.

### Electrical Properties Characterization

The resistance of LM ink before and after ultrasonic sintering was measured by a desktop multimeter (KEITHLEY DAQ7510). A lead screw platform (CL‐01A, Haijie Technology Co., Ltd) was exploited to provide constant force during tensile cycle process.

### Ultrasonic Sintering Under Water

The dome‐shaped bulk was printed by 3D printer (X400, Hori). LM ink was coated on the bulk surface and encapsulated by tapes. Copper wires related to the ink. The bulk in water was placed. An ultrasonic Homogenizer (JY92‐IIDN) was used to generate ultrasonic waves. As shown in Figure S17 (Supporting Information), ultrasonic horn was just above the bulk.

### Samples with Rough Surface

Samples with grooves on the surface were fabricated by 3D printing (P150, Boston Micro Fabrication). LM ink was coated on the surface and the grooves. Fluidity of LM ink allows it to fill the grooves. After that, ultrasonic horn was placed on the bottom of the sample to sinter the LM ink (Figure S18, Supporting Information). Ultrasonic power was 720 W.

### Ultrasonic Sintering on Sandpaper

The sandpaper was first adhered to an acrylic board (30 × 30 × 2.4 mm). The board was fixed on the titanium board as shown in Figure 5g. Ultrasonic horn was placed on the titanium board as shown in Figure 5g. Ultrasonic power was 200 W and ultrasonic time was 0.5 s. The frequency of ultrasound was 20 kHz. The rough surface will decrease the adhesion force between LM ink and the substrate, so the ultrasonic power to prevent the detach of LM ink was decreased.

### Surface Profile of Sandpaper

The sandpaper was adhered to an acrylic board (30 × 30 × 2.4 mm). After that the sample surface profile was measured by an Optical Profiler (Nexview NX2).

## Conflict of Interest

The authors declare no conflict of interest.

## Supporting information

Supporting Information

Supplemental Video 1

## Data Availability

The data that support the findings of this study are available from the corresponding author upon reasonable request.

## References

[1] a) B. Chen , Y. Cao , Q. Li , Z. Yan , R. Liu , Y. Zhao , X. Zhang , M. Wu , Y. Qin , C. Sun , W. Yao , Z. Cao , P. M. Ajayan , M. O. L. Chee , P. Dong , Z. Li , J. Shen , M. Ye , Nat. Commun. 2022, 13, 1206; 35260579 10.1038/s41467-022-28901-9 PMC8904466

[2] a) T. Lim , M. Kim , A. Akbarian , J. Kim , P. A. Tresco , H. Zhang , Adv. Healthcare Mater. 2022, 11, 2102382; 10.1002/adhm.202102382 35112800

[3] a) C. Dong , A. Leber , T. D. Gupta , R. Chandran , M. Volpi , Y. Qu , T. Nguyen‐Dang , N. Bartolomei , W. Yan , F. Sorin , Nat. Commun. 2020, 11, 3537; 32669555 10.1038/s41467-020-17345-8 PMC7363815

[4] a) H. Lu , G. Yun , T. Cole , Y. Ouyang , H. Ren , J. Shu , Y. Zhang , S. Zhang , M. D. Dickey , W. Li , S. Y. Tang , ACS Appl. Mater. Interfaces 2021, 13, 37904; 34319083 10.1021/acsami.1c09776

[5] a) G. Chen , H. Wang , R. Guo , M. Duan , Y. Zhang , J. Liu , ACS Appl. Mater. Interfaces 2020, 12, 6112; 31941273 10.1021/acsami.9b23083

[6] a) E. Palleau , S. Reece , S. C. Desai , M. E. Smith , M. D. Dickey , Adv. Mater. 2013, 25, 1589; 23334983 10.1002/adma.201203921

[7] a) Y. Zheng , Z. He , Y. Gao , J. Liu , Sci. Rep. 2013, 3, 1786;

[8] a) C. Pan , K. Kumar , J. Li , E. J. Markvicka , P. R. Herman , C. Majidi , Adv. Mater. 2018, 30, 1706937; 10.1002/adma.201706937 29405442

[9] a) C. W. Park , Y. G. Moon , H. Seong , S. W. Jung , J. Y. Oh , B. S. Na , N. M. Park , S. S. Lee , S. G. Im , J. B. Koo , ACS Appl. Mater. Interfaces 2016, 8, 15459; 27250997 10.1021/acsami.6b01896

[10] a) M. Zhang , G. Li , L. Huang , P. Ran , J. Huang , M. Yu , H. Yuqian , J. Guo , Z. Liu , X. Ma , Appl. Mater. Today 2021, 22, 100903;

[11] L. Tang , S. Yang , K. Zhang , X. Jiang , Adv. Sci. (Weinh) 2022, 9, 2202043. 35754311 10.1002/advs.202202043 PMC9376824

[12] G. H. Lee , H. Woo , C. Yoon , C. Yang , J. Y. Bae , W. Kim , D. H. Lee , H. Kang , S. Han , S. K. Kang , S. Park , H. R. Kim , J. W. Jeong , S. Park , Adv. Mater. 2022, 34, 2204159. 10.1002/adma.202204159 35702762

[13] S. Cheng , C. Hang , L. Ding , L. Jia , L. Tang , L. Mou , J. Qi , R. Dong , W. Zheng , Y. Zhang , X. Jiang , Matter 2020, 3, 1664.

[14] S. Liu , M. C. Yuen , E. L. White , J. W. Boley , B. Deng , G. J. Cheng , R. Kramer‐Bottiglio , ACS Appl. Mater. Interfaces 2018, 10, 28232. 30045618 10.1021/acsami.8b08722

[15] a) L. Tang , L. Mou , J. Shang , J. Dou , W. Zhang , X. Jiang , Mater. Horiz. 2020, 7, 1186;

[16] W. Lee , H. Kim , I. Kang , H. Park , J. Jung , H. Lee , H. Park , J. S. Park , J. M. Yuk , S. Ryu , J. W. Jeong , J. Kang , Science 2022, 378, 637. 36356149 10.1126/science.abo6631

[17] Z. Xu , L. Ma , J. Yan , S. Yang , S. Du , Composites, Part A 2012, 43, 407.

[18] Z. Lai , R. Xie , C. Pan , X. Chen , L. Liu , W. Wang , G. Zou , J. Mater. Sci. Technol. 2017, 33, 567.

[19] Z. Xu , Z. Li , Y. Qi , J. Yan , Ceram. Int. 2019, 45, 9293.

[20] L. Ma , Z. Xu , K. Zheng , J. Yan , S. Yang , Ultrasonics 2014, 54, 929. 24295911 10.1016/j.ultras.2013.11.005

[21] K. Yasui , Acoustic Cavitation and bubble dynamics, Springer, Switzerland 2018.

[22] T. J. Mason , J. P. Lorimer , Applied Sonochemistry, Wiley, Hoboken, NJ, USA 2002.

[23] I. D. Joshipura , H. R. Ayers , G. A. Castillo , C. Ladd , C. E. Tabor , J. J. Adams , M. D. Dickey , ACS Appl. Mater. Interfaces 2018, 10, 44686. 30532957 10.1021/acsami.8b13099

