# Supplementary material for: Ultrasonic‐Enabled Nondestructive and Substrate‐Independent Liquid Metal Ink Sintering
Source: Adv Sci (Weinh). 2023 Jun 14;10(23):2301292. doi: 10.1002/advs.202301292 (PMC10427386; doi:10.1002/advs.202301292)
Supplement: Supplementary file 1 — Supporting Information [file ADVS-10-2301292-s002.pdf]

ADVANCED SCIENCE
Open Access

Supporting Information

for *Adv. Sci.*, DOI 10.1002/advs.202301292

Ultrasonic-Enabled Nondestructive and Substrate-Independent Liquid Metal Ink Sintering

Sanhu Liu, Zhiwu Xu, Guoqiang Li, Zhengwei Li, Zihan Ye, Zirong Xu, Wenjun Chen, Dongdong Jin and Xing Ma**

Supporting Information

Sanhu Liu, Zhiwu Xu, Guoqiang Li, Zhengwei Li, Zihan Ye, Zirong Xu, Wenjun Chen, DongdongJin, and Xing Ma**

S. Liu, Z. Li, Prof. Z. Xu, Z. Xu

State Key Laboratory of Advanced Welding and Joining, Harbin Institute of Technology, Harbin, 150001, China

School of Materials Science and Engineering, Harbin Institute of Technology, Harbin 150001, China.

G. Li, Z. Ye, Dr. W. Chen, Dr. D. Jin

Sauvage Laboratory for Smart Materials,School of Materials Science and Engineering, Harbin Institute of Technology (Shenzhen), Shenzhen, Guangdong 518055, China

Prof. X. Ma

Sauvage Laboratory for Smart Materials,School of Materials Science and Engineering, Harbin Institute of Technology (Shenzhen), Shenzhen, Guangdong 518055, China

State Key Laboratory of Advanced Welding and Joining, Harbin Institute of Technology, Harbin, 150001, China

Email: xuzw@hit.edu.cn(Z. Xu); maxing@hit.edu.cn (X. Ma)

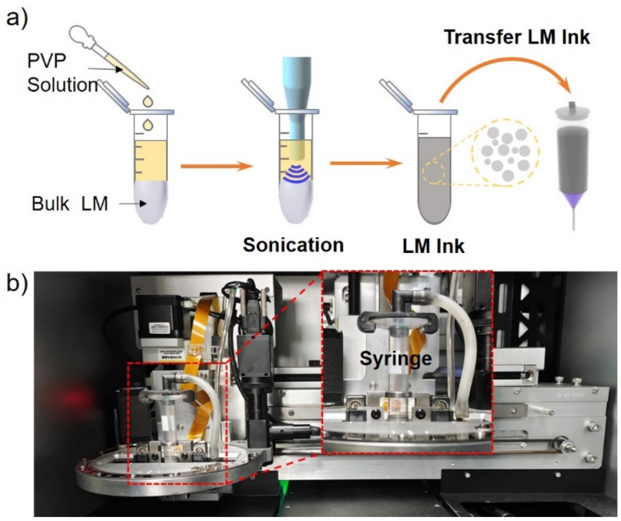

Figure S1.a) Scheme of LM ink preparation; b) Electronic printer for LM ink circuitprinting.

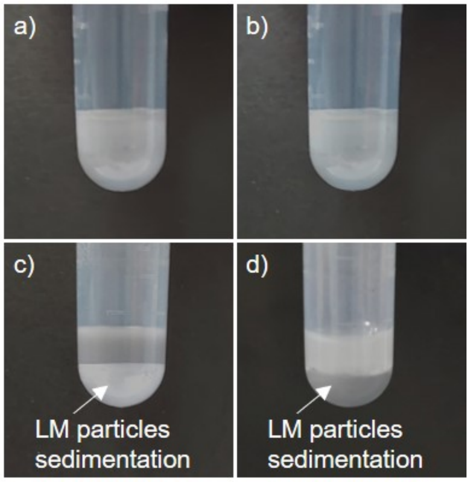

Figure S2. Influence of PVP on colloidal stability of LM particles ink suspension (ethanol). a) As-prepared fresh LM ink with addition of PVP. b) LM ink being settled for 12 h with addition of PVP. c) As-prepared fresh LM ink without PVP. d) LM ink being settled for 12 h without PVP. LM particles can maintain well dispersed state with addition of PVP. The LM ink here was prepared by sonication treatment, as shown in Figure S1. Without PVP, the LM ink was not stable and LM particles can easily aggregate.

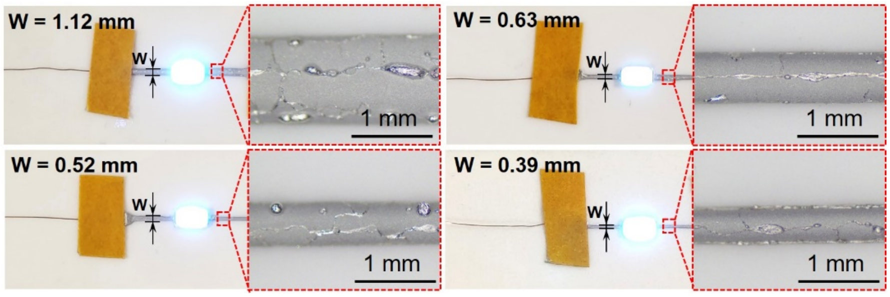

Figure S3. LM ink circuits with different line width (W) after ultrasonic sintering. The LED connected with LM ink circuits lighted on, proving that conductive paths were obtained inside the LM circuits.

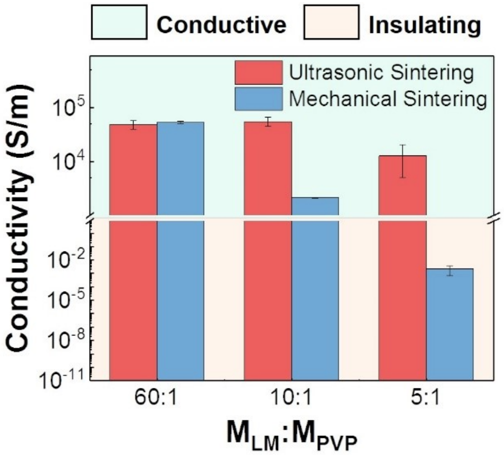

Figure S4. Conductivity of LM ink with different PVP content after ultrasonic sintering and mechanical sintering. The M_{LM} represents mass of LM and M_{PVP} represents mass of PVP. TheLMinkwas prepared by sonication treatment as Figure S1 shown. After printing LM ink line on the Al_2O_3 board (The line has the same shape as that shown in Figure 3a), ultrasonic sintering and mechanical sintering was applied to obtain conductive line. The length of the line was 10 mm. Ultrasonic power was 720 W and ultrasonic time was 2 s. (error bars: SD, n = 5)

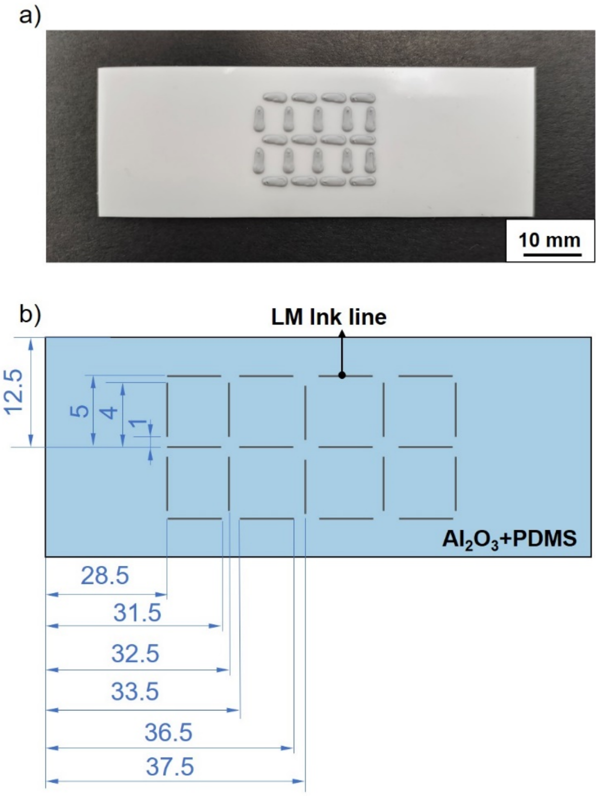

Figure S5. a) Photographof the LM ink linesprintedon anAl_2O_3 board. A layer of 60 μm PDMS film is placed between Al_2O_3 and the LM ink lines. b) Position details of the LM ink lines. The thickness of the Al_2O_3 board is 1 mm.

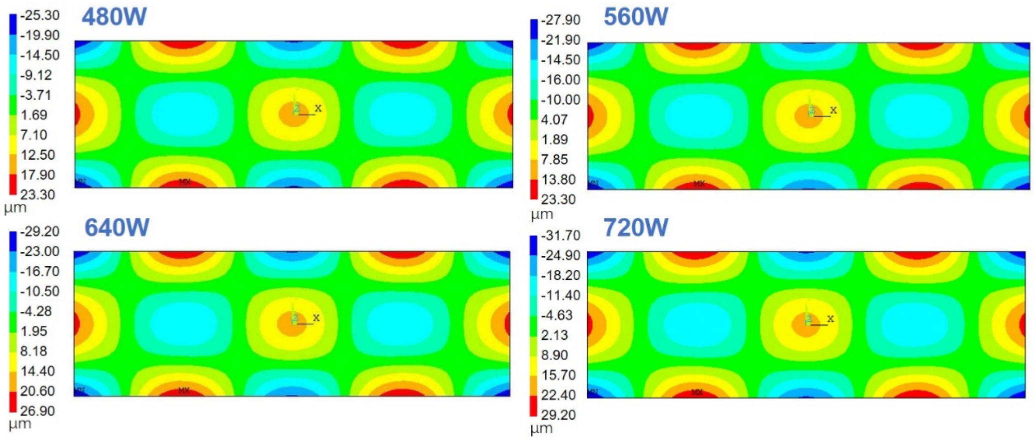

Figure S6. Ansys simulation of vibration amplitude distribution under different ultrasonic power. Thickness of the PDMS layer is much smaller than that of Al_2O_3. To simplify the calculation, the PDMS is neglected during simulation process.

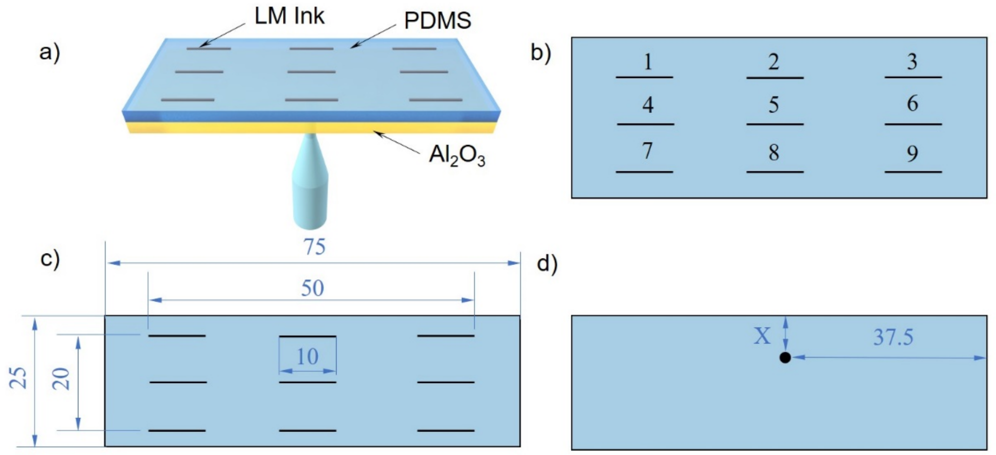

Figure S7. a) Scheme of the ultrasonic sintering process. b)Scheme of the 9 LM ink circuits. c) Position details of the 9 LM circuits. d) Position of the ultrasonic source (ultrasonic horn). X represents the distance from the horn to the edge of the board.

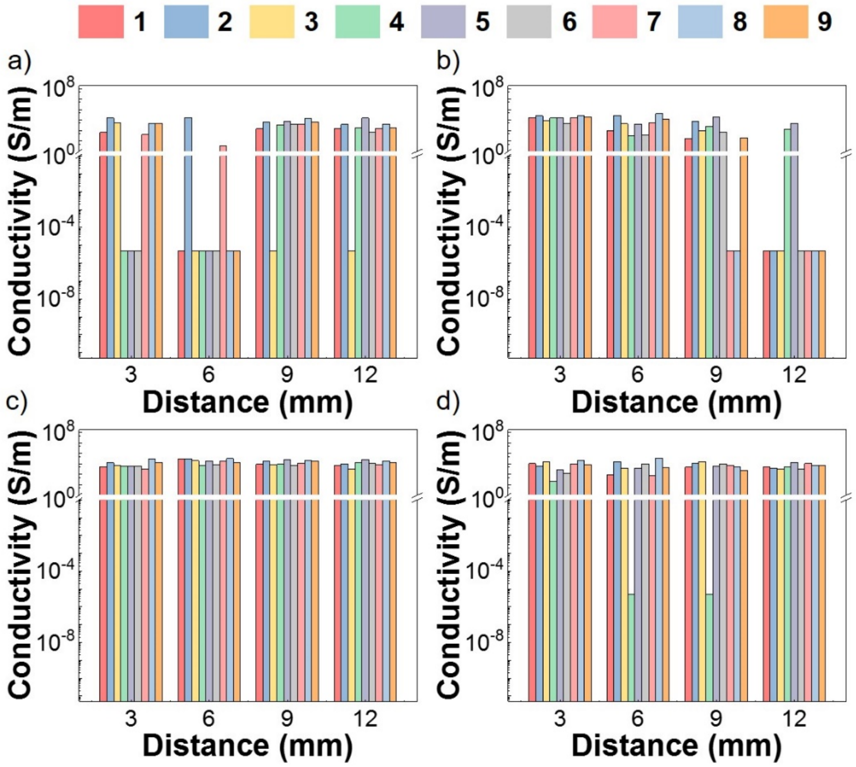

Figure S8. Average conductivityof LM ink under a) 480, b) 560, c) 640, d) 720 W ultrasonic power.Due to the fluctuation of data, conductivity of each sample was shown in Figure S7-10.

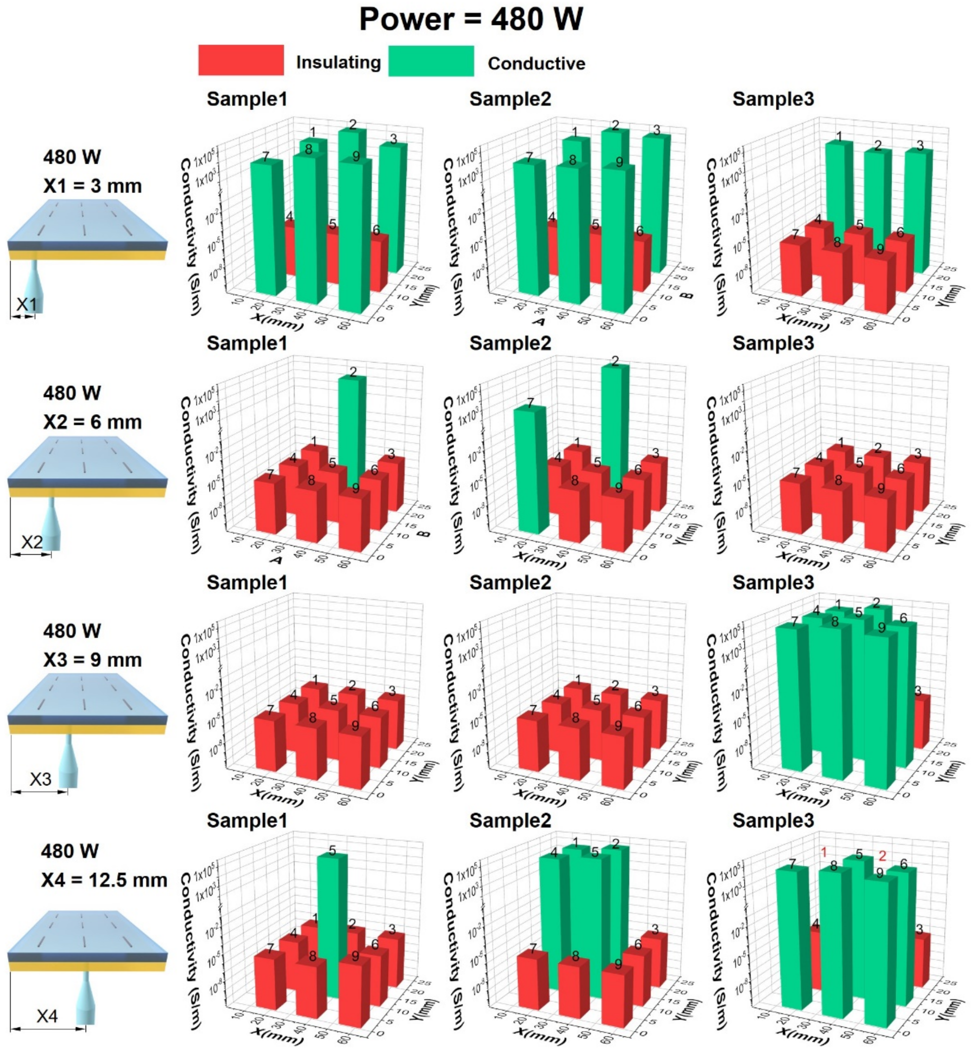

Figure S9. Conductivity variation with different ultrasonic position (480 W). 3 samples were prepared for each condition and conductivity of each sample was plotted.The label of each line was plot on the column. Some red columns were covered by green columns and we marked it with red numbers.

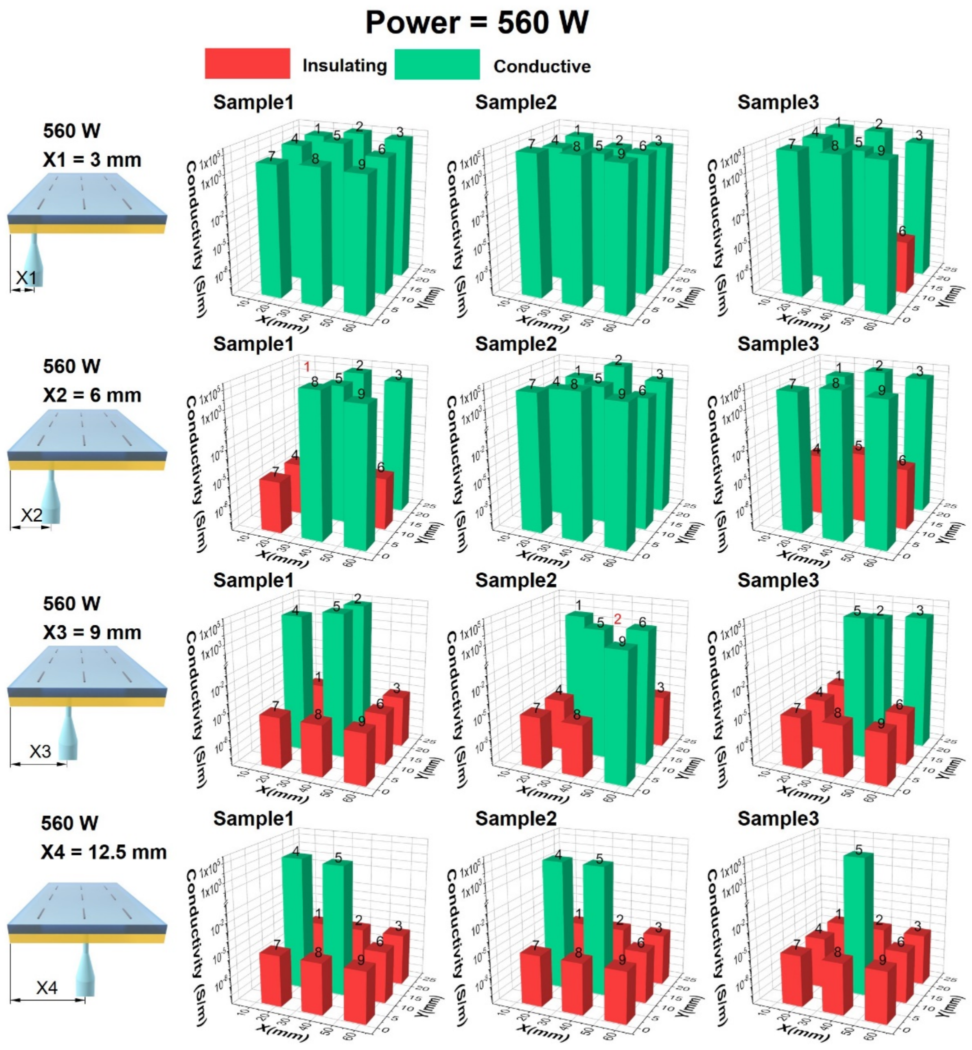

Figure S10. Conductivity variation with different ultrasonic position (560 W).3 samples were prepared for each condition and conductivity of each sample was plot. The label of each line was plot on the column. Some red columns were covered by green columns and we marked it with red numbers.

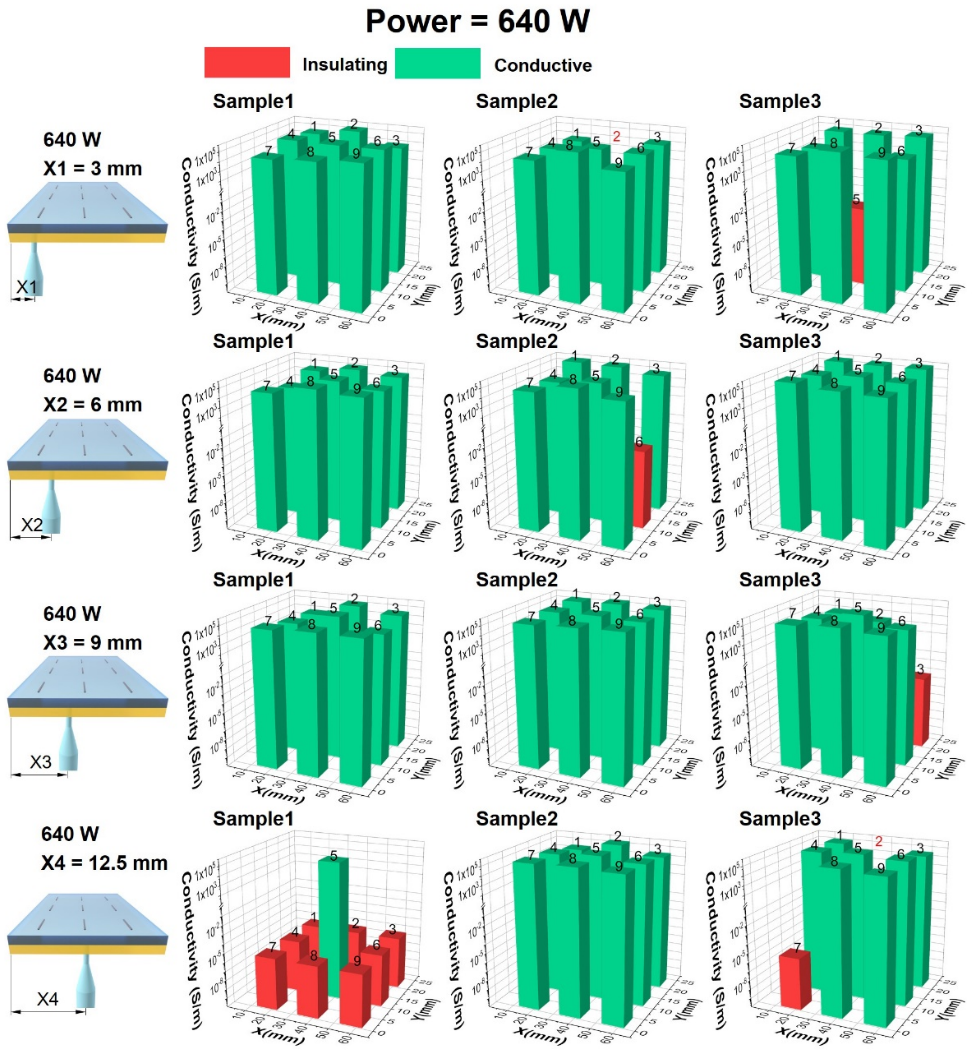

Figure S11. Conductivity variation with different ultrasonic position (640 W).3 samples were prepared for each condition and conductivity of each sample was plot. The label of each line was plot on the column. Some red columns were covered by green columns and we marked it with red numbers.

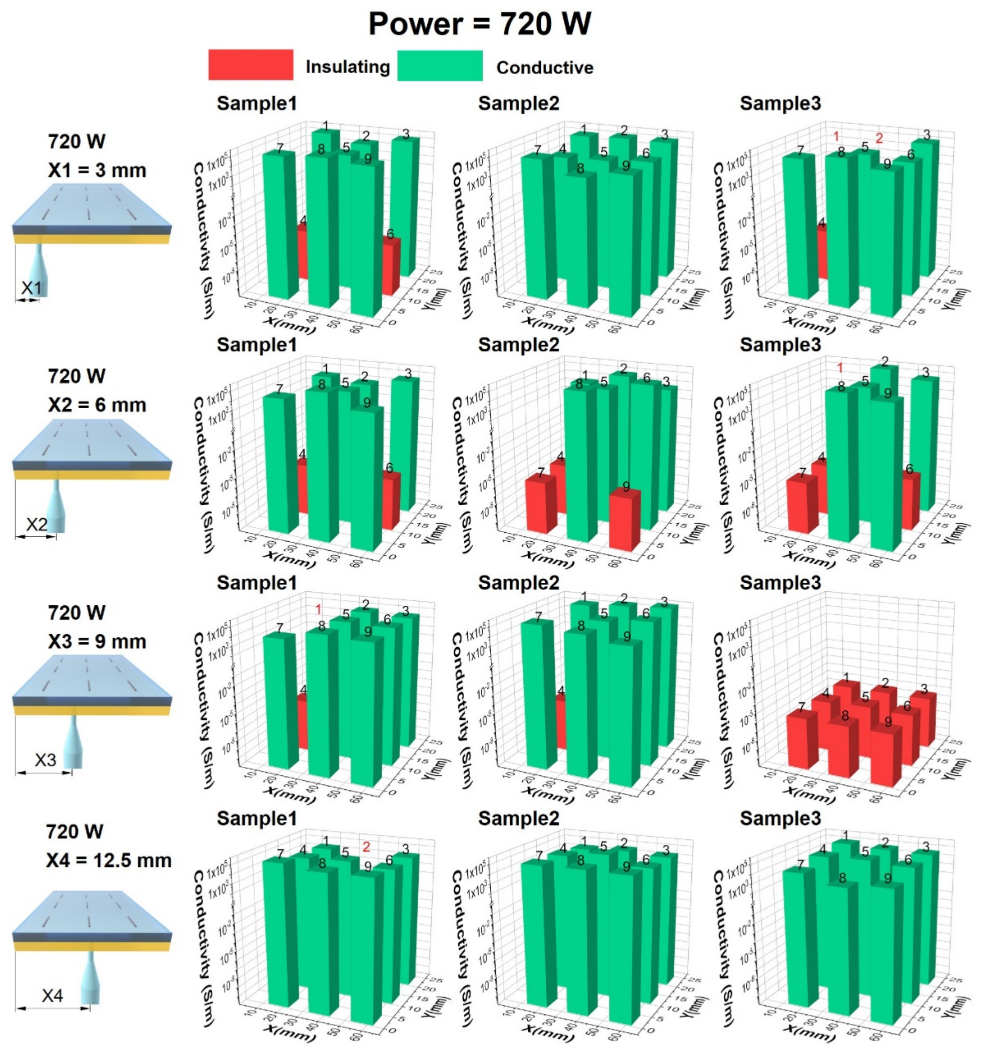

Figure S12. Conductivity variation with different ultrasonic position (720 W).3 samples were prepared for each condition and conductivity of each sample was plot. The label of each line was plot on the column. Some red columns were covered by green columns and we marked it with red numbers.

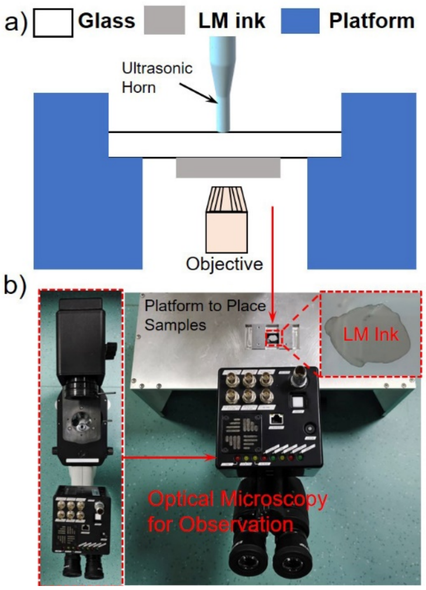

Figure S13. a) Scheme of LM ink pattern coated on aglass. b) Platform for real-time observation and video recording of the ultrasonic sintering process. Glass coated with LM ink pattern was fixed on a metal platform and an optical microscopy was placed under the platform for microstructure observation. The CCD in the microscopy record the change of LM ink pattern while applying ultrasound.

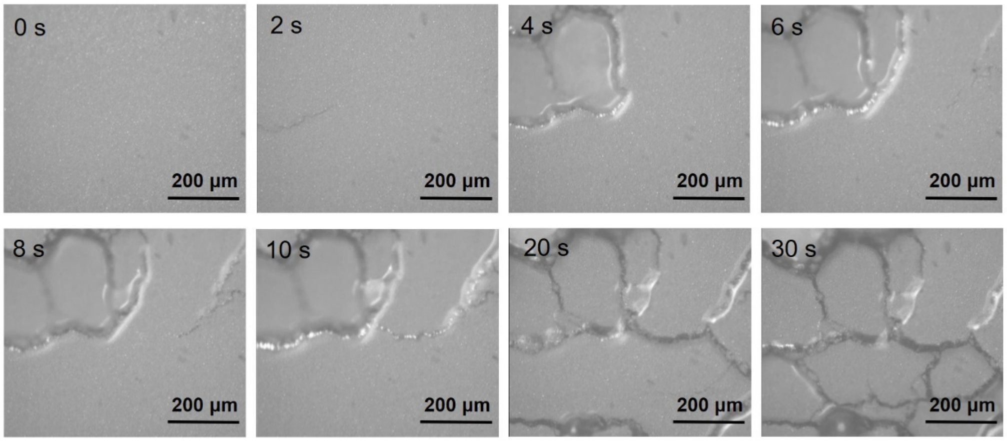

Figure S14. Snap-shoot images of LM ink pattern during the ultrasonic sintering process.

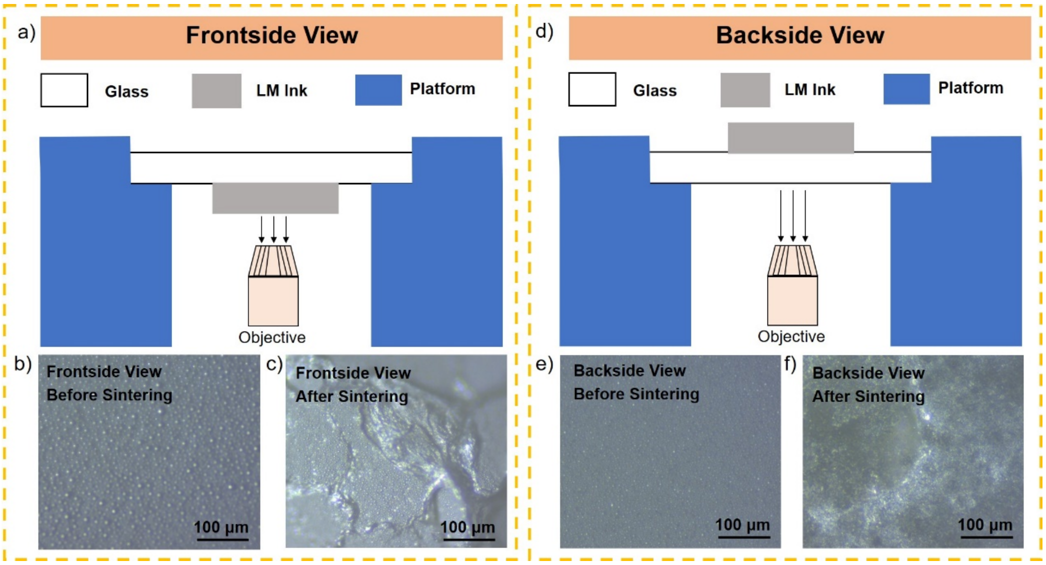

Figure S15. a) Scheme of frontside view of LM ink sample. Frontside view of the LM ink pattern b) before and c) after ultrasonic sintering. d) Scheme of backside view of LM ink sample. Backside view of the LM ink pattern e) before and f) after ultrasonic sintering.

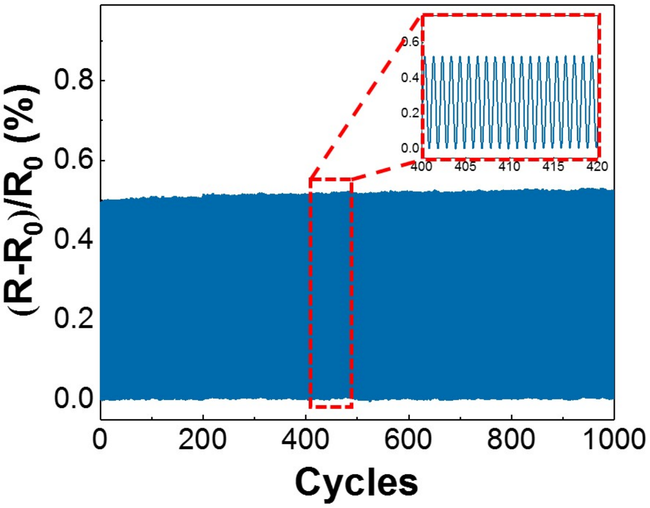

Figure S16. Relative resistance response of the pressure sensor under pressure during 1000 cycles, where the sensor is loaded with 100 g weights.

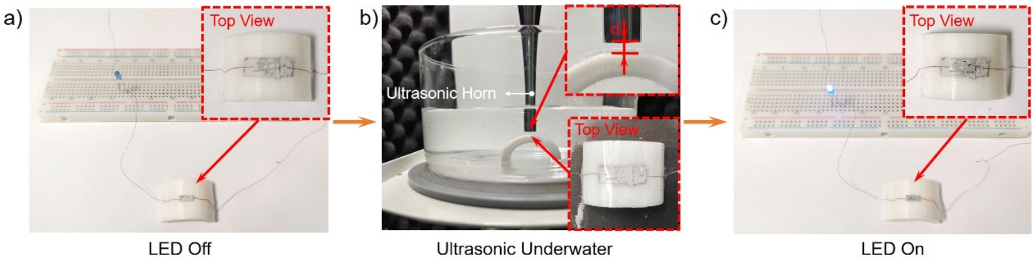

Figure S17. Ultrasonic sintering of a LM circuit under water.a) A dome-shaped sample holder was prepared by 3D printing and the LM ink circuit was directly coated on its top surface.b) Theholder was immersed in water and theultrasonic horn was placed on top of the LM circuit with certain distance (sintering distance: “d”). c) LM ink circuits were sintered and LED was on.

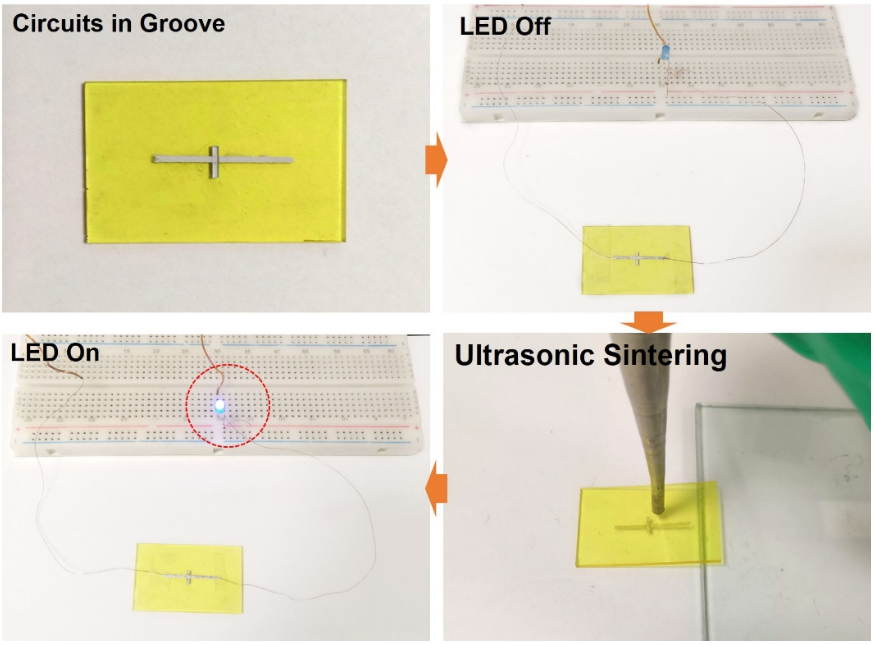

Figure S18. Ultrasonic sintering of LM ink pattern loaded within a groove on a rigid resin board (acrylic resin) that was prepared by a 3D printer (P150, Boston Micro Fabrication).

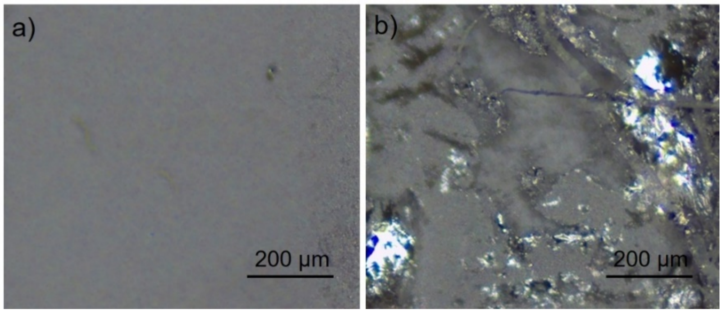

Figure S19. The micro-morphology of LM ink circuit on the rough surface shown in Figure 5e a) before and b) after ultrasonic sintering.Bright LM could be found after ultrasonic sintering, suggesting that LM particles were broken to fuse together and the circuits became conductive.

Table S1. Differences between Ref 16 and our work

	**Ref 16**<br>**Science, 2022, 378(6620): 637-641]**	**Our work**
**Material system**	➢ LM particles were immobilized in polymer matrix.<br>➢ The distance between liquid metal particles is relatively far.	➢ LM particles were not immobilized in polymer matrix.<br>➢ The distance between liquid metal particles is relatively close
**Sintering mechanism**	LM particles were connected by **LM particles network** due to the pressure provided by the cavitation.	Oxide film wrapped around LM particles was broken due to the vibration. **The LM inside was connected as whole part** rather than LM particles network.
**Research objective**	The as-prepared LM particle composites were used to **construct flexible electronics by sonication treatment in the water.**	Our research objective was to sinter LM ink by **applying ultrasound on various substrates** for the fabrication of flexible &printed electronics in **versatile applications scenarios.**
**Research significance**	LM particle network composites Promoted the development of **stretchable skin electronics** and highly **integrated stretchable electronics.**	➢ Ultrasonic sintering**preserved the original morphology** of LM circuits.<br>➢ LM ink Circuits were ultrasonic sintered on various substrates of **complex surface topography** that can hardly be realized by currently used mechanical sintering.
**Other contributions**		➢ Expand the ultrasonic sintering application.<br>➢ Investigate the influence of **ultrasonic power, ultrasonic position and vibration distribution** on board more specifically.<br>➢ Facilitate the realization of LM-based electronic applications in versatile practical scenarios.

Note S1

It should be noticed that not all sintering results follow the same rule. For example, when the ultrasonic power was 480 W and X = 9 mm, some circuits at the edge were sintered (Figure S7). On the contrary, circuits in other groups were not sintered. Such phenomenon could be induced by a variety of factors. During ultrasonic sintering process, oscillation of ultrasonic waves cannot be avoided. For an ultrasonic transducer which generates ultrasonic radiation, piezoelectric ceramic in combination with a vibration plate is used for high frequencies. Generally, piezoelectric materials in ultrasonic transducers vibrate with the frequency of an AC voltage applied to the materials. A piece of piezoelectric material vibrates most strongly when it is driven at its resonance frequency (f_0). Resonance frequency is determined by the mass and stiffness of a piezoelectric material. In other words, the resonance frequency is determined by the volume and shape of a material if the density of a material is kept constant. In addition, there are multiple resonance frequencies for a piece of material. Fluctuation of AC power frequency will also influence the vibration. Thus, the ultrasonic wave will change drastically with a slight change of the above factors, which influence the vibration distribution on Al_2O_3 board. Other factors are related to our board structure. Our simulation results are based on a 75 × 25 × 1 mm board. A different vibration distribution will appear when there is deviation of the board size. Besides, the interface between PDMS and Al_2O_3 generates extra reflection and attenuation of ultrasonic waves, which changes the resonance of ultrasonic waves on Al_2O_3 board. The combination of all the factors make our resistance results fluctuate to some extent. To clarify the complex mechanism and obtain accurate ultrasonic sintering results, further investigation is needed to analyze interface structure and ultrasonic waves resonance of different materials.

Besides, we conducted the sintering experiment shown in Figure S7 with 4 different ultrasonic powers (480 W, 560 W, 640 W and 720 W) corresponding to the results given in Figure S9, S10, S11, and S12 respectively. Under each power, we placed the horn at 4 different locations ("We used "X" to represent the distance between ultrasonic horn and the board's long side edge, which represent 4 locations, including X1 = 3 mm, X2 = 6 mm, X3 = 9 mm, X4 = 12.5 mm") as described in Figure S7. So, in total we have 16 different sintering conditions with specific power and position ("X") for each condition. Under each sintering condition, we preformed 3 duplicate samples named "Sample1", "Sample 2", "Sample 3". Fluctuation of frequency and attenuation of ultrasound will influence the sintering results. So, we can observe some inconsistence between 3 duplicate samples for each condition, which is quite normal for ultrasonic treatment. However, a general rule can be found out that with the increasing of ultrasonic power, the conductivity of all the samples increased, as indicated by many more "green" columns in Figure S11 and S12 as compared to Figure S9 and S10. In addition, the results suggest that we may adjust the location of the ultrasonic horn and repeat ultrasonic treatment in order to ensure sufficient sintering for future applications.
